# Virtual Reality Therapy for People With Epilepsy and Related Anxiety: Protocol for a 3-Phase Pilot Clinical Trial

**DOI:** 10.2196/41523

**Published:** 2023-01-24

**Authors:** Hannah Gabrielle Gray, Danielle Tchao, Samantha Lewis-Fung, Susanna Pardini, Laurence R Harris, Lora Appel

**Affiliations:** 1 Department of Psychology York University Toronto, ON Canada; 2 Department of Biology York University Toronto, ON Canada; 3 OpenLab University Health Network Toronto, ON Canada; 4 Department of General Psychology University of Padova Padova Italy; 5 Digital Health Lab, Centre for Health and Wellbeing Bruno Kessler Foundation Trento Italy; 6 School of Health Policy & Management York University Toronto, ON Canada; 7 Michael Garron Hospital Toronto, ON Canada

**Keywords:** epilepsy, anxiety, virtual reality, exposure therapy, eHealth, digital health, virtual reality exposure therapy, cognitive behavioral therapy, CBT, nonpharmacological intervention, biomedical technology

## Abstract

**Background:**

Anxiety is one of the most common psychiatric comorbidities in people with epilepsy and often involves fears specifically related to the condition, such as anxiety related to the fear of having another seizure. These epilepsy- or seizure-related fears have been reported as being more disabling than the seizures themselves and significantly impact quality of life. Although research has suggested that exposure therapy (ET) is helpful in decreasing anxiety in people with epilepsy, no research to our knowledge has been conducted on ET in people with epilepsy using virtual reality (VR). The use of novel technologies such as an immersive VR head-mounted display for ET in this population offers several benefits. Indeed, using VR can increase accessibility for people with epilepsy with transportation barriers (eg, those who live outside urban centers or who have a suspended driver’s license owing to their condition), among other advantages. In the present research protocol, we describe the design of an innovative VR-ET program administered in the home that focuses on decreasing anxiety in people with epilepsy, specifically anxiety related to their epilepsy or seizures.

**Objective:**

Our primary objective is to examine the feasibility of the study protocol and proposed treatment as well as identify suggestions for improvement when designing subsequent larger clinical trials. Our secondary objective is to evaluate whether VR-ET is effective in decreasing anxiety in a pilot study. We hypothesize that levels of anxiety in people with epilepsy will decrease from using VR-ET.

**Methods:**

This mixed methods study comprises 3 phases. Phase 1 involves engaging with those with lived experience through a web-based questionnaire to validate assumptions about anxiety in people with epilepsy. Phase 2 involves filming videos using a 360° camera for the VR-ET intervention (likely consisting of 3 sets of scenes, each with 3 intensity levels) based on the epilepsy- and seizure-related fears most commonly reported in the phase 1 questionnaire. Finally, phase 3 involves evaluating the at-home VR-ET intervention and study methods using a series of validated scales, as well as semistructured interviews.

**Results:**

This pilot study was funded in November 2021. Data collection for phase 1 was completed as of August 7, 2022, and had a final sample of 18 participants.

**Conclusions:**

Our findings will add to the limited body of knowledge on anxiety in people with epilepsy and the use of VR in this population. We anticipate that the insights gained from this study will lay the foundation for a novel and accessible VR intervention for this underrecognized and undertreated comorbidity in people with epilepsy.

**Trial Registration:**

ClinicalTrials.gov NCT05296057; https://clinicaltrials.gov/ct2/show/NCT05296057

**International Registered Report Identifier (IRRID):**

DERR1-10.2196/41523

## Introduction

### Background

Anxiety is the most common psychiatric comorbidity in people with epilepsy, with approximately 28% of the population suffering from at least one anxiety disorder, although different studies have reported varying prevalence [1]. In addition to decreasing quality of life and self-efficacy, anxiety may limit independence in people with epilepsy, who are already facing restrictions or limitations owing to other consequences of their neurological condition, such as suspension of their driver’s licenses [2]. People with epilepsy with comorbid anxiety also report more negative side effects from their antiseizure medications as well as poorer memory compared with those that do not have anxiety [2]. Moreover, anxiety may have a negative impact on seizure management; for example, individuals with idiopathic generalized epilepsy in particular have reported that sleep deprivation and stress are seizure triggers [3]. Yet, anxiety in people with epilepsy has received little research attention and is commonly not recognized nor treated [1,2].

Hingray et al [1] proposed 4 main types of anxiety disorders that are associated with epilepsy in adults: epileptic social phobia, seizure phobia, epileptic panic disorder, and anticipatory anxiety of epileptic seizures. The anxiety faced in people with epilepsy tends to stem from the uncertainty that is associated with the nature of the disorder. Moreover, people with epilepsy may fear places where they have previously experienced a seizure, as well as situations that could be physically dangerous should they have a seizure [1]. This anxiety can result in avoidance of situations that are crucial for independent daily living (such as taking public transit or a shower) [1]. In some cases, this anxiety may lead to obsessive behaviors and agoraphobia [1].

The underlying theoretical model of clinical anxiety implicates a series of cognitive and behavioral processes such as dysfunctional beliefs, classical conditioning, and operant conditioning [4]. In general, people with anxiety are characterized by two types of dysfunctional cognitions: (1) exaggerated estimates of the likelihood of negative consequences related to the feared condition and (2) exaggerated estimates of the severity of harm [5]. Over time, the fear becomes reinforced and may lead to conditioned behaviors in response to such stimuli [5]. To manage these manifestations of anxiety, individuals frequently adopt safety behaviors that reduce their feelings of fear in the short-term, such as avoiding the situation that leads to their anxiety [5]. Because of the short-term relief that they feel from these safety behaviors, individuals with anxiety often continue displaying these behaviors, which ultimately works to maintain or increase the anxiety associated with their feared situations [5]. These safety behaviors may impede everyday functioning and lead to a decreased quality of life as well as fewer opportunities for positive experiences, further impacting mood regulation [5-7]. For example, a safety behavior may include only leaving the house with trusted individuals [1,8]. However, it should be noted that in some cases, these behaviors may be warranted if they are required for seizure safety as indicated by a neurologist (ie, not considered safety behaviors fueled by anxiety) [1].

Exposure therapy (ET) is an evidence-based technique [9,10] that is helpful in facilitating the management of dysfunctional anxiety associated with a specific or generalized stimulus and associated safety behaviors [5]. Graded ET involves repeatedly exposing people to realistic situations that trigger their anxiety, often in a hierarchical manner [5]. ET targets feared stimuli that can be clustered in different categories. The categories may include situations that involve animals or specific human populations (such as spiders, people with HIV, and clowns), objects or specific spaces (eg, toilets, knives, and certain numbers), specific situations (such as driving, darkness, and feeling uncertain), thoughts and beliefs (eg, unwanted sexual thoughts, memories of traumatic events, and premonitions of untimely accidents), or interoceptive and physiological stimuli (eg, racing heart, feeling out of breath, and a skin blemish) [5].

Ultimately, ET aims to help individuals understand that they are capable of being in the situations that provoke their anxiety without needing to implement their safety-seeking behaviors that perpetuate avoidance behaviors [5]. By training individuals to overcome the thought patterns that cause their debilitating anxiety, ET may also help to interrupt the cycle of anxiety by disconfirming the individual’s misperceptions of their threats [5].

Virtual reality (VR) is a computer-simulated environment that allows users to feel as though they are in a different physical place, and it has been used as a tool to deliver ET for a variety of anxiety disorders. For example, VR-ET has previously been used to treat posttraumatic stress disorder, social anxiety disorder, and panic disorder [11]. VR has also been increasingly used as a nonpharmacological therapy for a variety of conditions, such as for people with dementia [12], including veterans with dementia [13]. Recent feasibility studies have also suggested that VR may be used to decrease anxiety in patients in the intensive care unit [14]. However, people with epilepsy have commonly been excluded from VR studies owing to the concern that using it may trigger seizures in people with photosensitive epilepsy. Although limited research is available on the use of VR in people with epilepsy, hesitations regarding the use of VR in this population have not been substantiated, and clinicians and researchers are increasingly considering VR for use in this population [15-17].

Some studies have highlighted that VR can complement imaginal and in vivo exposures [18,19], which are often otherwise difficult to implement. For instance, limitations of imaginal exposures include that individuals may try to avoid thinking about the scene that makes them anxious or they may struggle to imagine the scene accurately [5]. As VR elicits realistic sensory-motor experiences, using VR to deliver ET may be more successful than imaginal exposures for individuals who have difficulty mentally conjuring up distressing scenes or for individuals who try to avoid thinking about them [20,21]. A further limitation of in vivo exposure is treatment adherence; users commonly drop out before completing the recommended number of sessions. As VR-ET does not place individuals in the actual physical environment, participants have the opportunity to learn how to cope with the feared situation in a less threatening environment [22]. Thus, VR-ET has the potential to increase individuals’ willingness to do in vivo exposures and decrease avoidance in the real physical environment because their preparedness makes the natural stimuli appear less overwhelming [23].

Research has suggested that including ET as a part of treatment for anxiety may also be helpful for people with epilepsy [24,25]. To our knowledge, no research has been conducted to date on ET in people with epilepsy using VR. In this population specifically, using an immersive VR head-mounted display (HMD) for ET has several advantages over traditional therapies. For example, research not specific to epilepsy has illustrated that VR-ET is especially useful when it is impractical to do exposures in person, such as during the COVID-19 pandemic [26]. Even outside of the pandemic, using VR to deliver this customizable therapy limits the need for travel, thereby removing a barrier for people with epilepsy because driver’s licenses are typically suspended for 6 months to 1 year after a confirmed seizure. Moreover, using VR for ET offers the potential for significant time and cost savings compared with traditional ETs, in addition to increasing equitable access to mental health resources for those outside of urban centers [27].

### Study Overview and Objectives

We are designing and evaluating a VR-ET program specifically for people with epilepsy and anxiety in a pilot clinical trial (Trial Registration: ClinicalTrials.gov NCT05296057) that will be divided into 3 phases. Phase 1 will involve engaging with those with lived experiences through a web-based questionnaire. The aim of phase 1 will be to validate assumptions about anxiety specifically related to epilepsy or seizures. Videos of scenes that are most commonly reported as generating anxiety in people with epilepsy will then be filmed using a 360° camera in phase 2. In phase 3, these VR exposure scenarios will be piloted by people with epilepsy in a clinical trial.

Table 1 represents a visual overview of the study methodology. This table was designed using the template provided by the Standard Protocol Items: Recommendations for Interventional Trials. It outlines the assessments at various time points: T0 (baseline), T1.1 (first day of the VR-ET intervention), T1.12 (the twelfth and last day of the VR-ET intervention, depending on each participants’ progress that may be extended to T1.13 or T1.14), and T2 phases (1 week after the last exposure session).

The primary objective of this pilot clinical trial is to examine the feasibility of the study protocol, determine effect sizes, and identify suggestions for improvement when designing a subsequent larger clinical trial. The secondary objective is to evaluate whether VR-ET reduces epilepsy-related anxiety in people with epilepsy.

**Table 1:** Overview of phases 1 to 3, including enrolment, virtual reality exposure therapy (VR-ET) intervention design and delivery, data collection, and outcome measures.

			Phase 1		Phase 2		Phase 3				
							T0^a^		T1.1^b^-T1.12^c^		T2^d^
Phase 1 informed consent			✓								
Phase 3 informed consent							✓				
Eligibility screen							✓				
Design and film VR^e^ scenes					✓						
VR-ET intervention									✓		
**Assessments**											
	Interview					✓				✓	
	Phase 1 questionnaire	✓									
	Background questionnaire					✓					
	VR Induced Symptoms and Effects					✓					
	Diagnostic protocol proposed by Hingray et al [1]					✓					
	Epilepsy Anxiety Survey Instrument					✓				✓	
	Perceived Stress Scale					✓				✓	
	Subjective Units of Distress Scale							✓			
	Fast Motion Sickness Scale							✓			
	Igroup Presence Questionnaire									✓	
	System Usability Scale									✓	

^a^T0: baseline.

^b^T1.1: first day of the VR-ET intervention.

^c^T1.12: The twelfth and last scheduled day of the VR-ET intervention. Depending on each participants’ progress, the intervention may be extended to T1.13 or T1.14.

^d^T2: 1 week after the final exposure session.

^e^VR: virtual reality.

## Methods

### Ethics Approval

The study was approved by ClinicalTrials.gov (ID: NCT05296057) on March 25, 2022, and was fully approved by the York University Human Participants Review Committee on May 31, 2022 (certificate number: 2022-105). Amendments to Phases 2 and 3 were approved by the York University Human Participants Review Committee on September 15, 2022.

### Data Collection and Storage

Any digital data, such as responses to web-based questionnaires, will be uploaded and saved to a secure server. Any hard copy documents will be stored in a locked cabinet. Both digital data and hard copy documents will be retained for 10 years after the end of the study and then securely destroyed.

### Phase 1

#### Phase 1 Overview

Very little research currently exists on the specific epilepsy-related fears that people with epilepsy experience. As such, the first phase of this study will aim to validate assumptions about the most common epilepsy-related fears in this population so that the videos recorded in phase 2 are reflective of fears that are relevant for exposure in phase 3. To gather these data, phase 1 consists of a web-based survey delivered over the Qualtrics platform to 2 groups of participants: (1) people with epilepsy and (2) people that are affected by epilepsy but do not have epilepsy themselves (eg, through a family member, as a personal caregiver, as a health professional, or if they work with people who have epilepsy).

#### Phase 1 Recruitment

The investigators are using a diverse recruitment strategy. First, we are leveraging our connection with relevant community associations, namely Epilepsy Toronto. Epilepsy Toronto is an association that works to support people with epilepsy and their loved ones. Epilepsy Toronto has agreed to send an advertisement on their email listserv as well as post the study advertisement on their social media accounts. Second, a snowballing strategy will be used wherein participants will be prompted at the end of the questionnaire to share the link with others in their network. Phase 1 had a target sample size of 15 participants, including individuals with epilepsy and individuals affected by epilepsy.

#### Phase 1 Eligibility

Individuals aged 18 years and older who have epilepsy or are affected by epilepsy (such as through a family member that has epilepsy) may participate in this study. Phase 1 does not have any exclusion criteria.

#### Phase 1 Consent

The phase 1 informed consent form appears as the first page of the Qualtrics questionnaire and requires a digital signature. The Qualtrics questionnaire is configured such that participants cannot progress to the questionnaire without first providing explicit consent.

#### Phase 1 Protocol

Both groups of participants are asked to list up to 5 scenes (ie, locations, situations, or environments) that generate epilepsy-related anxiety in people with epilepsy, based on their personal experience. Participants are then asked to describe the scene that causes the greatest amount of anxiety in more detail. If the participant chooses, they may describe other scenes that they listed in more detail as well.

The questions included in the phase 1 questionnaire were selected and phrased so that they guide the filming of the 360° videos in phase 2 to resemble the anxiety provoking scenes as realistically as possible (Textbox 1). Analysis of phase 1 responses may identify ways in which the phrasing of questions may be improved in a later study to increase the realism of the exposures and thus the efficacy of the intervention.

**Overview of the questions asked in the phase 1 questionnaire (version completed by individuals with epilepsy). Individuals affected by people with epilepsy completed a version with alternate wording appropriate to their perspective.**

1. Demographics:
  - Year of birth
  - Sex at birth
  - Describe the type of epilepsy or seizures you have and the symptoms that you experience during a seizure. *The intention of asking this question is to determine if the symptoms that the person with epilepsy experiences during a seizure (such as losing consciousness, muscle control, etc) influences their anxiety.*
2. Scenes:
  - List up to 5 scenes (such as situations, locations, or environments) where you feel anxiety related to your epilepsy or seizures. Next to the open-ended spaces to list each scene, participants are asked to rank their avoidance behavior from the following options:
    Avoid completely
    Often avoid
    Sometimes avoid
    Do not avoid (but still feel anxious)
  - To ensure that the 360° videos, which will be recorded in phase 2, are realistic and resemble the scenes from the person with epilepsy’s point of view as closely as possible, participants are asked to provide detail about the scene that causes the greatest amount of anxiety, as if they were designing a scene for a movie from their perspective. Participants are prompted to do so by asking:
    What do you see, hear, and feel?
    What are you and others doing in this scene?
    What in particular about this scene makes you anxious?
    What changes to this scene would (1) increase and (2) decrease your anxiety? *Since ET often involves exposing people to their fears in a hierarchical manner, the VR-ET program will involve exposing participants to levels of the scenes reported in phase 1. Although subject to change based on phase 1 results, exposures will have 3 levels. This question about how changes to the scene may increase or decrease anxiety will help determine the different films that should be recorded to represent the fear.*
  - Participants are given the option of describing additional scenes.
  - Closed-ended questions: Participants are provided with a series of scenes developed based on the researchers’ knowledge of epilepsy or seizure-related anxiety, as well as evidence from the literature [1,25] and asked to rate how likely these scenes would generate epilepsy- or seizure-related anxiety. The rating options are as follows: strongly agree, somewhat agree, somewhat disagree, strongly disagree, and prefer not to say. The specific scenes are listed below. Ratings on these scenes will also help to inform phase 2 recordings.
    On a subway platform
    On a subway train
    On a bus
    On stairs or elevated platforms
    In a washroom or other place with hard surfaces
    At a shopping center
    Completely alone
    With a few people that I know (but not well)
    With a few people that I have a close relationship with
    At a large social gathering or party where I only know a few people
    At a large social gathering or party where I know some or all people
    Surrounded by complete strangers in a public setting
    Going for a walk outside
    Exercising with gym equipment
  - Is there anything else you’d like to share about your epilepsy- or seizure-related anxiety?

#### Phase 1 Data Analysis

Sections 2A-2C and 2E of the phase 1 questionnaire (Textbox 1) will be coded by 2 researchers and then analyzed using the constant comparison method. The purpose of this analysis will be to determine which scenes were most commonly reported in the open-ended questions and the attributes that the virtual environment will reflect when they are recorded in phase 2. Section 2D of the phase 1 questionnaire (Textbox 1) will be analyzed using descriptive statistics, including calculating the frequencies of each discrete variable.

### Phase 2

#### Phase 2 Protocol

**Figure 1:**
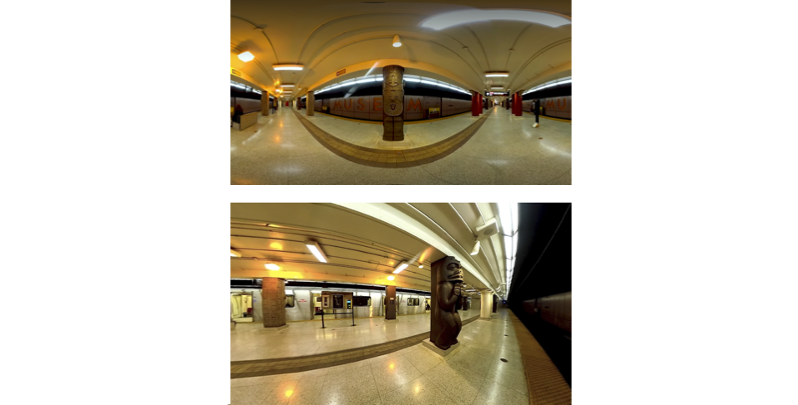
Examples of virtual reality (VR) scenes that may be shown during the VR exposure therapy. Top: Toronto subway station with a few people; equirectangular frame from a 360° VR film. Bottom: A subway that has arrived at a Toronto station with no people around; 360° VR snapshot.

Phase 2 will involve the development of the minimal viable product VR intervention. Although the design of the exposure hierarchies may be subject to change based on results from phase 1, we aim to record 3 sets of scenes in phase 2. Each set will have 3 scenes of different intensity levels that are designed to generate increasing amounts of anxiety (Figure 1). The scenes will be recorded using a 360° camera. Using 360° video is a cost-effective way to design photorealistic virtual environments with potential to create an increased sense of presence during exposure sessions when compared with computer-generated graphics [28,29]. An example of a scene that was recorded with a 360° camera can be seen [30] (click and drag on the video to scroll in 360°).

Participants will then be exposed to the 3 scenes of the set that most closely resemble their own epilepsy-related fears in phase 3 using the VR HMD. In summary, it is expected that phase 2 will involve filming nine 360° videos (3 sets × 3 scenes each). When filming these videos, we will ensure to the best of our abilities that any possible sensory triggers for people with epilepsy, such as common visual or auditory triggers, will not be included in the videos.

Phase 2 will not involve any data analysis.

#### Phase 2 Recruitment

Phase 2 does not include any participants and only involves members of the research team. Specifically, the research team will record the 360° videos, and those who sign release forms will be actors in the videos.

The researchers will ensure that while recording the videos, any bystanders who happen to be present at the scene are at least 2 m away from the lens of the VR 360° camera. At a distance of 2 m, the resolution of a 360° video is typically too low for faces to be identifiable, and so the privacy of bystanders will be maintained. Only members of the research team involved in the recordings who have signed release forms will be within 2 m of the 360° camera, as appropriate for the scene. Researchers who will be interacting with participants face to face in phase 3 (over a video call or in person) will not be included as actors in the videos.

### Phase 3

#### Phase 3 Overview

**Figure 2:**
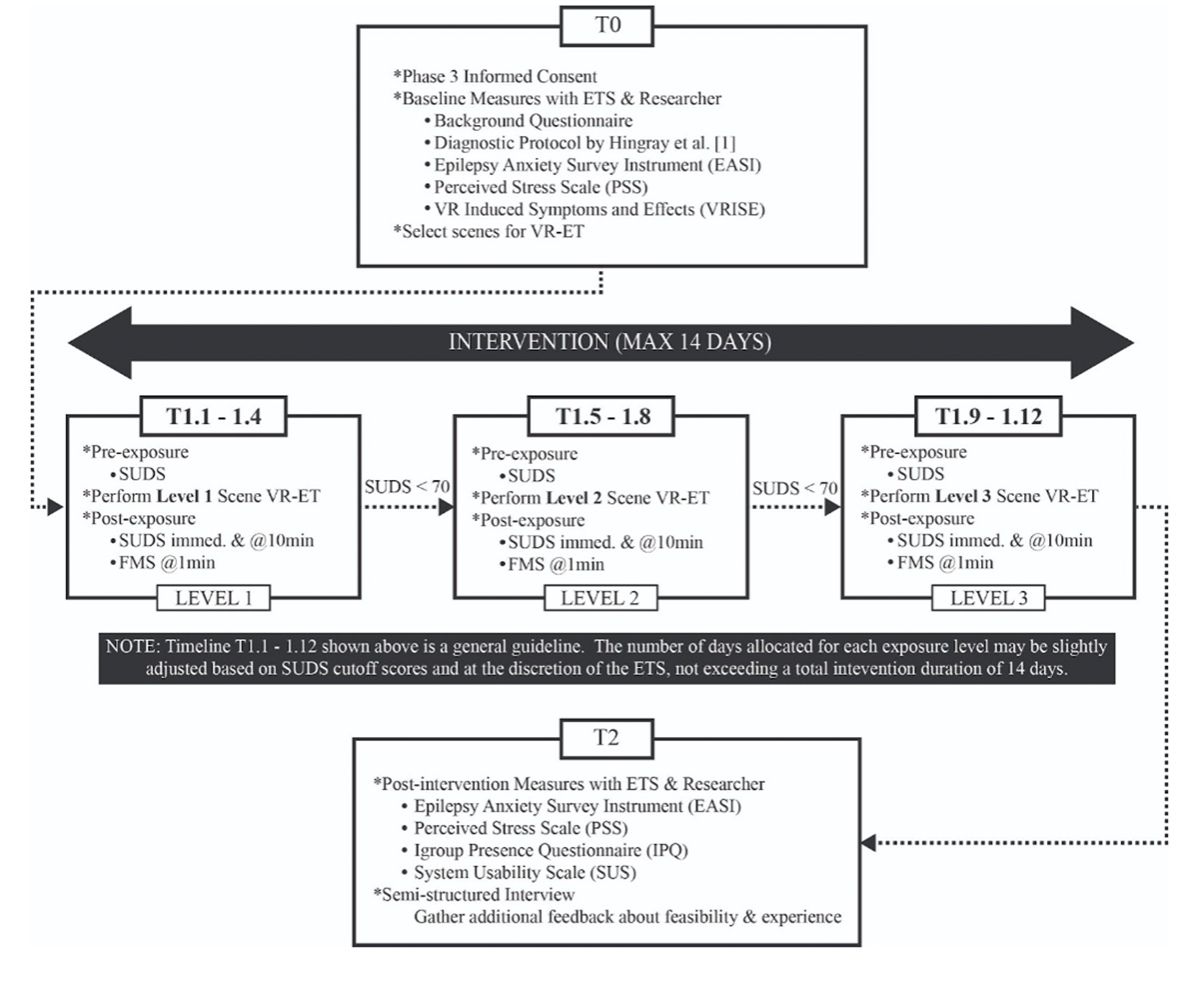
Overview of the schedule for phase 3. ETS: exposure therapy specialist; FMS: Fast Motion Sickness Scale; SUDS: Subjective Units of Distress Scale; VR-ET: virtual reality exposure therapy.

Participants in phase 3 will undergo the VR-ET intervention program, which will take place over approximately 2 weeks (Figure 2). Before beginning the program, participants will have an interview with an ET specialist (ETS) where they will discuss which set of scenes recorded in phase 2 fits best with their greatest anxieties. Participants will also respond to several questionnaires and assessments. Each participant will begin with the lowest exposure level (in other words, the scene that generates the least amount of anxiety) and gradually work their way up to the highest exposure level (which is the scene that generates the greatest amount of anxiety). Ideally, each exposure level will be performed every day for approximately 4 days, after which the participant will move onto the next level. Each day that they complete an exposure, participants will provide a score on the Subjective Units of Distress Scale (SUDS) at 3 specific time points. To help determine the efficacy of VR-ET, participants will have a second interview and repeat certain assessments 7 days after completing the ET program. It is hypothesized that VR-ET will help participants to habituate to their fears and ultimately lead to decreased anxiety.

#### Phase 3 Recruitment

Phase 3 will use the same recruitment methods as phase 1, including advertising through Epilepsy Toronto and using a snowball sampling strategy. In addition, phase 1 participants will be given the opportunity at the end of the questionnaire to enter their email address if they wish to receive more information about participating in phase 3.

Our target sample size for phase 3 is 5 people with epilepsy (people that do not have epilepsy themselves will not be included in phase 3). A sample size of n=5 was determined to be appropriate as this pilot study is evaluating feasibility of VR-ET for this population without aiming to determine clinical efficacy. In addition, sample size calculations are not necessary for feasibility studies according to the Consolidated Standards of Reporting Trials guidelines [31].

#### Phase 3 Consent and Eligibility

##### Overview

Participants will answer an eligibility screening after providing digital explicit consent through the phase 3 informed consent form (delivered over the Qualtrics platform). The eligibility screening will be completed over a telephone or video call with a member of the research team to help create rapport.

Even if the participant does not meet any of the exclusion criteria, they may be excluded from the study at any point. For example, if the research team is concerned that the treatment may have more negative consequences than positive for any reason, the principal investigator will consult with the ETS or make an executive decision to withdraw the participant from the study. However, information collected up until withdrawal may be (anonymously and in aggregate) included in the study analysis and findings. Participants are made aware of this in the phase 3 informed consent form.

##### Phase 3 Inclusions

Individuals with diagnosed epilepsy aged 18-65 years with self-reported mild or moderate epilepsy-related anxiety may participate in this study.

##### Phase 3 Exclusions

ET works by increasing anxiety in the short-term for the purpose of decreasing anxiety in the long-term. Because it is expected that participants’ anxiety will rise during the ET sessions, anyone who was told by a neurologist or personally believes that stress has provoked their seizures in the past will be excluded from phase 3. Anyone with a self-reported diagnosis of panic disorder or self-reported severe epilepsy-related anxiety will also be excluded from this pilot study. However, should VR-ET for people with epilepsy become a standard treatment, people whose seizures are triggered by stress should decide with their medical team if the benefits of this treatment outweigh the risks. Therefore, people with epilepsy should consider whether a long-term decrease in stress from VR-ET (and a consequent decrease in seizures triggered by stress) may be worth the short-term increase in stress and the accompanying risk of seizures.

Those who have been told by a neurologist that they have photosensitivity or that they have had photoparoxysmal responses during an electroencephalogram will also be excluded from phase 3 [15]. Excluding people with photosensitivity from the study is not expected to significantly impact the sample size because only approximately 3% to 5% of people with epilepsy have photosensitive seizures [32]. However, even individuals who were never told by a neurologist that they have had photosensitive seizures but believe that photosensitivity may contribute to their seizures will also be excluded from phase 3 [15]. Similarly, individuals who previously experienced seizures after being in a VR environment will also be excluded from this study.

As depression and anxiety are common in people with epilepsy [2], many people that are interested in participating in the study may be taking psychotropic medications, such as antidepressants. Because antidepressants may take up to 12 weeks to show their full effects, it would be unclear whether improvements in anxiety from baseline are due to a response to medication or VR-ET [33]. Similarly, drugs such as benzodiazepines, which are used to treat seizures, may act to suppress the anxiety felt by people with epilepsy during VR-ET [34]. Therefore, to better evaluate the impact of VR-ET alone, people who began taking antidepressants, benzodiazepines, or medical marijuana within 12 weeks of phase 3 will be excluded.

Although individuals will not be excluded from the study if they have been taking benzodiazepines for longer than 12 weeks before starting phase 3, they will be asked to avoid taking them before the exposure session (only if possible and if it does not interrupt their antiseizure medication schedule). This is because benzodiazepines have immediate effects and may therefore artificially lower participants’ SUDS scores [35]. A complicating factor in this regard is that some people with epilepsy are prescribed drugs such as benzodiazepines as a rescue medication and may therefore use them to stop or prevent seizures [34,36]. In clinical practice, it is possible that the patient may take a benzodiazepine before, during, or after performing an exposure solely out of fear that they will have a seizure (and not necessarily because they are actually about to have a seizure). In this case, the patient would be actively avoiding the anxiety that is associated with the exposure. By using avoidance, the exposure’s usefulness is nullified because the patient is shielded from feelings of fear. They also momentarily lose the opportunity to learn that they are capable of handling these feelings of anxiety.

Other phase 3 exclusion criteria include individuals who have tonic-clonic seizures more than once a month, individuals who have functional seizures, individuals who cannot speak or understand English, individuals with open wounds on their face, and individuals with cervical conditions or injuries that would make it unsafe for them to use an HMD.

We will not be excluding individuals with memory deficits, as memory issues are common in people with epilepsy [37]. It is not a concern that memory difficulties will prevent participants from using the VR device properly as they will always be on a video call with a member of the research team who can guide the participant in using the VR headset as necessary.

#### Phase 3 Protocol

##### Baseline (T0)

###### Baseline Interview

Before beginning the VR-ET intervention, participants will have a one-on-one interview with an ETS over a telephone or video call. In this interview, the ETS will explain the 3 sets of scenes that were recorded in phase 2. Then, through discussion, the ETS and participant will determine which exposure set fits best with their individual epilepsy-related fears.

After choosing the appropriate exposure set, the ETS and participant will discuss the most appropriate order in which the 3 scenes associated with the set should be delivered during the intervention. As ET is typically delivered in a hierarchical manner, participants will be exposed to the scenes in the order of least anxiety provoking to most anxiety provoking. The order of exposure scene delivery will be individualized for each participant based on their unique experiences and fears. For example, the exposure set that fits best with 2 participants (hereinafter referred to as participants A and B) may be a shopping mall with its associated social aspects. In this case, the 3 levels may be scenes of a shopping mall with crowds of different sizes (eg, scenes with 2, 10, and 30 people). Participant A may have a fear of not being around many people in a public setting, such as a shopping mall, because it could mean that less help would be available in case they were to get a seizure. Therefore, participant A would first be exposed to the mall scene with 30 people, then 10 people, and then finally 2 people. In contrast, participant B may have a fear of being around many people in a public setting because they worry that having a seizure is embarrassing and would prefer to be surrounded by fewer people if they were to have a seizure [1]. Thus, the ETS would decide with participant B that they should first be exposed to the mall scene with 2 people, then 10 people, and finally 30 people. In summary, opportunities for tailoring the therapy will be built into the intervention through the (1) selection of scenario (location or theme), (2) selection of scenes (based on specific fears within a location such as the number of people in the scene), and (3) ordering of the scenes.

The aim of ET is to expose individuals to their fears until they habituate to the anxiety and learn to predict the outcome of their feared event as less anxiety provoking than they anticipated. Therefore, it is expected that participants may experience anxiety in anticipation of the exposure, during the exposure, and for a short period after completing the exposure. Although ET typically involves letting anxiety subside without instituting coping mechanisms, special considerations must be taken when working with the epilepsy population. Specifically, stress is a commonly self-reported seizure trigger for some people with epilepsy [38]. Although people with epilepsy for whom stress is a known or suspected seizure trigger will be excluded from phase 3, there is still a risk that stress may provoke a seizure in participants. To help prevent this, a number of protocols will be set in place (Textbox 2).

**Protocols put in place in case participants become too anxious before, during, or soon after the exposure sessions.**

**Coping mechanisms**

- Before the baseline interview, participants will be sent a document that provides anxiety coping mechanisms, namely a mindfulness strategy, a self-compassion strategy, and a meditative technique. Participants will be encouraged to practice these coping mechanisms before the interview as they will then review them with the exposure therapy specialist.
- Before each exposure, the researcher will remind participants that, if they feel too overwhelmed during the exposure, they should remove the virtual reality head-mounted display even if they have not completed the full exposure and use the coping mechanisms that they prepared with the exposure therapy specialist. (If the participant stops the exposure early and uses a coping mechanism, the researcher will make a note of this. Postsession Subjective Units of Distress Scale (SUDS) scores will be recorded regardless.)
- If the participant scores >70 on any of the SUDS tests after completing the exposure, whether they ended the exposure early or not, they will be instructed by the research member on the video call to use the coping mechanisms. (The remaining SUDS scores will still be recorded.)

**Additional strategies**

- If the participant’s anticipatory anxiety SUDS score (ie, the SUDS score that the participant records before performing the exposure) is >70 on the first day of beginning a new exposure level (eg, level 2), participants will still be encouraged to attempt the new level if their 10-minute postexposure SUDS score the previous day for the lower level (level 1) was <70.
- Even though we are excluding individuals who experience panic attacks, there is a risk that participants may panic before, during, or after the exposure. Therefore, members of the research team will be trained by a clinical neuropsychologist on how to identify when someone is having a panic attack and reach out to an on-call health professional.

###### Assessments

At baseline, the participant will also complete several assessments (Table 1 and Textbox 3), specifically the background questionnaire (Textbox 4); proposed diagnostic protocol for epilepsy-specific anxiety disorders [1]; Epilepsy Anxiety Survey Instrument (EASI); Perceived Stress Scale (PSS); and Virtual Reality Induced Symptoms and Effects (VRISE) assessment.

**Assessments at T0.**

**Background questionnaire**

- This questionnaire was created by the authors of this paper to be conducted at the baseline interview (Textbox 4). Its main purpose is to collect phase 3 participants’ demographic information and information about their history with anxiety and epilepsy.

**Epilepsy Anxiety Survey Instrument (EASI) [39]**

- The EASI is validated for assessing epilepsy-related anxiety features and severity. Note that by using the EASI, we are simultaneously using the brief Epilepsy Anxiety Survey Instrument (brEASI) [39]. The brEASI is a validated screening tool for anxiety disorders in people with epilepsy and is made up of 8 items that are already asked in the EASI. The brEASI is based on the fifth edition of the Diagnostic and Statistical Manual of Mental Disorders [40].

**Perceived Stress Scale**

- The Perceived Stress Scale assesses how an individual perceives their own levels of stress.

**Proposed diagnostic protocol for epilepsy-specific anxiety disorders [1]**

- Hingray et al [1] suggested a diagnostic enquiry for 4 proposed anxiety disorders specific to epilepsy: anticipatory seizure anxiety, seizure phobia, epileptic social phobia, and epileptic panic disorder. They also suggested an assessment for symptom severity of each of these disorders and an evaluation of avoidance behaviors.
  Note: On the basis of responses to the assessment by Hingray et al [1], individuals who appear to have epileptic panic disorder according to a clinical neuropsychologist will be excluded from the study.

**Virtual Reality Induced Symptoms and Effects (VRISE) assessment [41]**

- The VRISE assessment is one of the 4 domains included in the Virtual Reality Neuroscience Questionnaire and evaluates the intensity of motion sickness. The 3 other domains included in the Virtual Reality Neuroscience Questionnaire (user experience, game mechanics, and in-game assistance) are not relevant to our study. A lower score on the VRISE assessment suggests greater motion sickness.
  Note: Participants who score less than 25 on the VRISE assessment after virtual reality training during T0 will be withdrawn from the study.

**Overview of the questions to be asked in the Background Questionnaire (T0).**

1. Demographics
  - Contact information of the participant and their emergency contact
  - Year of birth
  - Sex assigned at birth and gender
  - Address that the participant will be doing the video calls from
2. Technology use
  - Previous experience with virtual reality (if any)
  - Comfort level on a scale of 1-10 with computers, smart phones, video call technology (specifically Zoom), virtual reality headsets
  - Low vision
  - Recent eye surgery
3. Epilepsy
  - Type(s) of seizures
  - Date of epilepsy diagnosis
  - Sensory triggers
  - Epilepsy safety protocol that should be set in place in case the participant were to have a seizure during the video call
4. Anxiety
  - Presence of anxiety as a side effect from any antiseizure medications currently taken
  - Anxiety disorder diagnoses. Participants will be asked to list any anxiety disorders that they were diagnosed with and the dates of diagnoses
  - History of experiencing preictal or postictal anxiety
  - Previous or current cognitive behavioral therapy (CBT). *If yes*:
    - Date the CBT program began
    - Date the CBT program was completed (or the expected completion date)
    - The helpfulness of the CBT program
    - If the CBT program is focused on anxiety. *If yes*, if it involved targeting epilepsy-related anxiety
    - If the CBT included exposure therapy
  - If the participant has previously undergone or if they are currently undergoing any exposure therapy. *If yes*, they will be asked similar questions as 4D (i-iv)
5. Medical marijuana and medication use: (As this question and others in the study may be sensitive and yet important for evaluating the impact of the intervention, all outcome measures will be prefaced with an explanation of the importance of answering honestly and to the best of participants’ abilities, as well as a reminder that participation is voluntary and that questions may be skipped without repercussion.)
  Current antiseizure medication(s)
  Current medical marijuana use
  Current antidepressants or medications to treat anxiety

###### Equipment Setup

The only in-person aspect of T0 will be when a member of the research team visits the participant’s residence to set up the VR equipment (Oculus Quest 2) and teaches them how to use and store the device. In teaching participants how to use the VR HMD, participants will practice using the device set to a neutral scene that is not expected to provoke anxiety in people with epilepsy according to phase 1 or existing literature. After using the VR HMD, participants will complete the VRISE questionnaire (Textbox 3) to record their baseline motion sickness tendencies. Note that anyone who scores below 25 on VRISE after using VR with a neutral scene will not be allowed to continue with the study.

At this time, the researcher will review the overall layout of the experiment and arrange a schedule with the participant for the exposure sessions, which will take place daily over one-on-one video calls. Ideally, the video calls (and therefore, exposure sessions) will take place at approximately the same time each day. Indeed, exposures should be performed at similar times each day to limit inconsistent and extraneous reasons for increased baseline anxiety levels.

##### Exposure Session Protocol (T1)

###### Overview

The VR-ET intervention will consist of an exposure session every day for up to 14 days over a one-on-one video call with a researcher. During each exposure session, the participant will be seated in a chair at their home. Exposures will be performed for approximately 5 minutes, which is lower than the recommended 10-minute threshold for mitigating motion sickness [42]. Each day that the participant performs an exposure, they will complete assessments: SUDS 3 times; the Fast Motion Sickness Scale; and, although not a formal assessment, participants will be asked about the frequency in which they used coping mechanisms during the exposure session and in the 10-minute period afterward (Textbox 5).

**Assessments at T1.**

**Subjective Units of Distress Scale (SUDS)**

- The purpose of collecting SUDS scores will be to quantitatively assess:
  1. How participants’ anxiety in anticipation of performing the exposure compares to their anxiety immediately after completing the exposure
  2. If their anxiety decreases over time after completing an exposure session (ie, immediately after the exposure vs 10 minutes after the exposure)
  3. If their overall anxiety progressively decreases over the course of each exposure level
- Each day that they perform an exposure, the participant will record a SUDS score at 3 time points:
  - Before putting on the head-mounted display and beginning the exposure. This measures the anxiety that the participant feels in anticipation of performing the exposure.
  - Immediately after completing the exposure
  - 10 minutes after completing the exposure

**Fast Motion Sickness Scale [43]**

- The purpose of collecting Fast Motion Sickness Scale scores will be to quantitatively measure motion sickness, specifically the general discomfort and nausea components, that participants may have experienced during the virtual reality exposure session. Participants will complete this assessment shortly after completing the first postexposure SUDS.

**Frequency of coping mechanism use**

- After completing the first postexposure SUDS test, participants will be asked how often, if at all, they used the coping mechanisms provided during the exposure (Textbox 2). After completing the second postexposure SUDS test, participants will be asked again about how often, if at all, they used the coping mechanisms since performing the exposure. The purpose of collecting this information is to understand whether differences in the use of coping mechanisms is related to the efficacy of the virtual reality exposure therapy intervention.

The purpose of having a researcher on a video call with the participant during exposure sessions is 3-fold. First, it provides a safety plan in case participants were to have a seizure during the exposure. This was put in place not because it is expected that participants might be triggered by the VR-ET but rather because people with epilepsy are prone to seizures, and therefore, it is important to have a seizure protocol in place. In the background questionnaire, the participant will provide details such as their emergency contact’s information, if they would like the researcher to call their emergency contact or an ambulance if they have a seizure (some people with epilepsy prefer an ambulance not to be called), and if they would prefer for the researcher to keep their video on for safety or to turn it off for privacy. Second, if the participant experiences panic, the researcher will contact an on-call health professional. Third, through discussion with a clinical neuropsychologist, being in someone’s presence (even virtually) while performing an exposure acts as a behavioral commitment from the individual who is receiving the treatment. Therefore, the participant will be less likely to display avoidance behaviors in relation to performing the exposure.

###### Exposure Session Timeline

Each participant will begin with the exposure scene that generates the least amount of anxiety and gradually work their way up to the scene that provokes the most anxiety. Each exposure scene will be performed daily for approximately 4 days, after which the participant will move onto the next level. However, if SUDS scores are >70 and at the discretion of the ETS, the current scene may be repeated until the participant is ready to progress. If a participant’s SUDS score is too high to begin the first level, they will be exposed to a different set and its associated scenes, even if it does not fit with their own anxiety as closely.

As such, the overall schedule may vary for each participant based on their individual progress. The participant is expected to complete the intervention in 12 days but will be allowed up to 14 days if additional days are required to complete a scene. If a participant has a seizure at any point during the 12 to 14 days that they are receiving the intervention, the VR-ET will be discontinued as a precautionary measure. However, data provided up until that point may still be used in analyses and they will participate in T2.

##### Postintervention (T2)

One week after completing the VR-ET intervention, the participant will answer several assessments (Table 1 and Textbox 6), specifically EASI; PSS; Igroup Presence Questionnaire (IPQ) [44]; and the System Usability Scale (SUS) [45]. The proposed diagnostic protocol for epilepsy-specific anxiety disorders [1] will not be repeated at T2 and will instead inform some of the open-ended questions that will be asked in the semistructured exit interview that will take place over telephone or video conferencing (Textbox 6). The interview will be conducted either by an ETS or a member of the research team and will aim to gather additional feedback about the participants’ experience. For example, participants will be asked to provide feedback on their experiences with the various devices, the subjective impact of the therapy, and what may be improved about the program and ET scenarios. Similar to T0, the only in-person aspect of T2 will take place when a member of the research team goes to the participant’s residence to collect the VR equipment.

**Outcome measures at T2.**

Repeat: Epilepsy Anxiety Survey Instrument and Perceived Stress Scale

**Igroup Presence Questionnaire [44]**

- The Igroup Presence Questionnaire assesses the subjective experience of being in a virtual environment when one is physically situated in another

**System Usability Scale [45]**

- The System Usability Scale assesses usability of the hardware and software

**Semistructured Exit Interview**

- To collect feedback (preferences and suggestions for improvement) on the:
  Virtual reality system usability and training
  Virtual reality exposure scenes
    - (ie, how realistically the videos simulated real-world scenarios that provoke anxiety for the participant)
  Intervention impact on anxiety
  Treatment delivery and format
  General experience

#### Phase 3 Data Analysis

As both quantitative and qualitative data will be collected in phase 3, a mixed methods approach will be used. Quantitative statistical analyses will be performed using the MiniTAB statistical package [46] and SPSS software (version 27.0; IBM Corp) [47]. To investigate the normal data distributions of the dependent variables, ranges of skewness and kurtosis will be determined. The Kolmogorov-Smirnov and the Shapiro-Wilk tests will also be performed to evaluate statistical significance (mean, 95% CI) and normality of the distributions. In addition, Cronbach *α* will be performed for each self-report questionnaire’s subscales. To explore sociodemographic features, frequencies, means, and standard deviations will be calculated.

To explore the effects of VR-ET, data collected at T0 and T2 will be compared. Clinically significant effects of the intervention will be investigated using the Jacobson-Truax method, based on the measures’ clinical cut-offs (Table 2). Moreover, SUDS scores (before the exposure, immediately after the exposure, and 10 minutes after the exposure) collected every day of T1 will be compared with each other. Participants’ SUDS scores that are recorded immediately after the exposure may be lower than their anticipatory anxiety levels, and if so, this may encourage the participant to do the next exposure. We will also consider our study to be successful if participants’ SUDS scores remain high in the first postexposure SUDS test but decrease at the 10-minute postexposure SUDS test. However, the study may still be deemed successful with unchanging SUDS scores if participants have reduced anxiety and engage in fewer safety-seeking behaviors after completing VR-ET, as based on T2 findings.

Using thematic analysis, qualitative responses to assessments and interviews will be assessed by the research team and then reported as frequencies using the procedure outlined by Braun and Clarke [48]. The constant comparison method will be used to analyze the responses to the open-ended questions asked at T0 and T2 [49].

**Table 2:** Clinically significant scoring for the phase 3 assessments.

		Items	Range	Scores	Citation
**Assessments**					
	Epilepsy Anxiety Survey Instrument	18	0-54	Higher scores suggest more severe anxiety	Scott et al [39]
	Brief Epilepsy Anxiety Survey Instrument	8	0-24	≥7 suggests a likely anxiety disorder	Scott et al [39]
	Perceived Stress Scale	14	0-56	Higher scores indicate greater perceived stress levels	Cohen et al [50]

## Results

This study was funded by a joint Junior Faculty Funds and Minor Research Grant from the York University Faculty of Health in November 2021. Recruitment for phase 1 was completed as of August 7, 2022, and had a final sample of 18 participants. Results for phase 1 will be published once data collection and analysis have been completed; data collected as of August 7, 2022, may be requested by contacting the corresponding author.

We are leveraging discussions with experts in the field to design the first VR-ET program specific to people with epilepsy which have led to some minor amendments (Textbox 7) to our original protocol.

**Summary of study amendments to improve the study’s methodology.**

- Phase 1 questionnaire
  - *Updated* the wording and layout so that it would be faster, simpler, and more intuitive for participants to fill out.
  - *Added* relevant questions, such as the specific prompts for each scene.
- Phase 1 informed consent form
  - *Updated* the wording to remove any language that may have overstated the risks of filling out the questionnaire.
  - *Adapted* it to be deliverable over a web-based platform.
- Phase 2 recordings
  - *Updated* the guidelines so that common sensory triggers for seizures will be avoided. This was added after discussion with an epileptologist about the use of virtual reality in people with epilepsy.
- Phase 3 assessments
  - *Added*: Epilepsy Anxiety Survey Instrument, Igroup Presence Questionnaire; System Usability Scale.
  - *Removed*: Beck Anxiety Inventory; Generalized Anxiety Disorder-7 scale.
- Honorarium
  - *Added* for phases 1 and 3.

## Discussion

### Principal Findings

Although the primary anxiety treatments for people with epilepsy are cognitive behavioral therapy and pharmacological treatments, minimal research has investigated anxiety treatments specifically for this population [51,52]. Moreover, few studies have investigated the use of ET for treating anxiety in people with epilepsy [24,25]. No studies to our knowledge have delivered ET to people with epilepsy using VR, despite VR-ET’s efficacy in other populations and the benefits that it could offer to people with epilepsy [11].

The main amendments made to the protocol included changes to the phase 3 anxiety assessments, after consultation with a clinical neuropsychologist who specializes in treating people with epilepsy. Indeed, when assessing anxiety in people with epilepsy, it is important to use validated assessments that do not inquire about physical manifestations of anxiety that may be confused with consequences of the neurological disorder. For example, the Beck Anxiety Inventory has been shown to be potentially less accurate in measuring anxiety in people with epilepsy because it contains items that reflect antiseizure medication side effects and symptoms of epilepsy, as opposed to symptoms of anxiety [39]. The Generalized Anxiety Disorder-7 scale was removed because it focuses on generalized anxiety disorder rather than anxiety in general or epilepsy-specific fears. Instead, the EASI was added.

The IPQ and SUS were also added to assess the feasibility of the intervention as well as provide validated feedback on participants’ experiences with the technology in preparation for the subsequent larger clinical trials. For example, the IPQ will help assess participants’ sense of presence in the virtual scene, which is important in VR-ET so that it resembles the real-life scenes that participants fear as closely as possible [18]. The IPQ will also be useful for determining if phase 2 was successful in realistically capturing the scenes. The interview at T2 will expand on responses to the IPQ as participants will be asked to provide verbal feedback on how future studies could improve the realism of the VR-ET scenarios, as well as the sense of presence that they generate. Furthermore, the SUS will provide useful information for optimizing procedures for training participants on how to use the VR equipment. The SUS may also help to identify if there were greater difficulties for people with cognitive deficits and inform how we could design a system that is accessible for all potential individuals with epilepsy. To assess motion sickness tendencies and symptoms after the VR-ET sessions, the VRISE and Fast Motion Sickness Scale were also included.

### Limitations

Pilot studies play a key role in the development or refinement of new interventions, assessments, and other study procedures, with the primary role of a pilot study being to examine the feasibility of a research endeavor [53]. Although the nature of a pilot study allows for a small sample size, we acknowledge that this is a limitation of both phases 1 and 3 and that, as a result, caution should be applied to the generalization of the findings. The sample sizes were determined based on resource constraints (funding and time) and are appropriate for a feasibility study that does not aim to assess clinical efficacy. To help account for the small sample size of phase 1, the baseline interview of phase 3 will act as an extension of phase 1 where we will gain more insight into the specific epilepsy- and seizure-related fears that people with epilepsy experience.

Another limitation to the generalizability of our findings is that phase 3 does not include a control group. Participants will be recruited to a known, albeit experimental, study and assessments will be conducted in an unblinded fashion. The choice to run an open-label pilot with no control group was intentional. Our primary goal is to report on the feasibility of recruitment, intervention design and implementation, as well as participant retention. Given the novelty of this initiative and our resources (funding and time), the research team agreed that this was the most appropriate way to systematically gather and incorporate user feedback before evaluating VR-ET on a larger scale. Future studies with larger sample sizes, a control group, and randomization will be necessary to evaluate clinical efficacy.

We acknowledge that it may not be feasible in clinical practice for therapists to have daily communication with their clients or patients. However, we are designing the intervention this way purposefully for the pilot study to maximize safety in case of a seizure or concerning psychological distress, such as a panic attack. In addition, as discussed with a clinical neuropsychologist, it will maximize the chances of therapeutic success if participants are in communication with someone, such as a therapist or researcher, while doing the exposures to help them form a behavioral commitment. Because this is a pilot study, it aligns more with the purpose of the research to maximize the chances of therapeutic success rather than to assess the feasibility of the proposed intervention within a professional setting. However, the ability to implement self-guided sessions in a controlled and safe virtual environment is an advantage of VR therapy. Therefore, future studies should investigate participants’ adherence to VR-ET and its efficacy when they perform the exposures daily without a researcher or therapist present.

Another limitation is that while we ask participants to avoid taking a benzodiazepine before performing the exposure and after their exposure until their anxiety settles, we cannot enforce this request because benzodiazepines may be used to treat seizures [34]. As previously discussed, taking a benzodiazepine can limit the effectiveness of the ET. It may be worthwhile to conduct future clinical trials in a safe and well-monitored environment, such as a hospital epilepsy monitoring unit, where participants are restricted from taking a benzodiazepine as much as possible. In this scenario, the participants would be in a safe environment in case they were to have a seizure and potential confounding effects would be reduced.

In conclusion, we are proposing a novel VR-ET as a treatment specific to people with epilepsy and their unique fears relating to their condition. VR is an especially promising tool for ET broadly as it allows for tailored exposure and control over the visual and auditory stimuli. When considering people with epilepsy, VR-ET offers additional advantages for this population. For example, given that the therapy can be administered in the comfort of one’s home, it increases accessibility for individuals with restricted independence and ability to travel. Overall, this study will contribute significantly to the fields of VR, ET, anxiety, and epilepsy and lay the groundwork for a more equitable therapy program for this population.

## Abbreviations

EASI: Epilepsy Anxiety Survey Instrument
ET: exposure therapy
ETS: exposure therapy specialist
HMD: head-mounted display
IPQ: Igroup Presence Questionnaire
PSS: Perceived Stress Scale
SUDS: Subjective Units of Distress Scale
SUS: System Usability Scale
VR: virtual reality
VRISE: Virtual Reality Induced Symptoms and Effects

## Data Availability

Data sharing is not applicable to this study as no data sets were generated or analyzed. The data collection tools (eg, surveys) created for this study are available from the corresponding author on reasonable request.
